# Active transport by cytoplasmic dynein sustains dendritic MAP2 localization in developing neurons

**DOI:** 10.1016/j.jbc.2026.113407

**Published:** 2026-08-07

**Authors:** Yoji Yonemura, Yuri Sakai, Rinaho Nakata, Ayaka Hagita-Tatsumoto, Tomohiro Miyasaka, Hiroaki Misonou

**Affiliations:** 1 Laboratory of Ion Channel Pathophysiology, Graduate School of Brain Science, Doshisha University, Kyotanabe-shi, Kyoto, Japan; 2 Department of Neuropathology, Faculty of Life and Medical Sciences, Doshisha University, Kyotanabe-shi, Kyoto, Japan; 3 Center for Research in Neurodegenerative Diseases, Doshisha University, Kyotanabe-shi, Kyoto, Japan

**Keywords:** microtubule-associated protein (MAP), neuron, dynein, localization, transport

## Abstract

MAP2 has been widely used as a marker of neuronal dendrites because of its extensive restriction in the somatodendritic region. Despite that, how the precise localization of such a soluble protein is established and maintained against diffusion has been elusive and long remained a mystery in neuroscience. In this study, using GFP-tagged MAP2 expressed in cultured hippocampal neurons, we discovered a crucial protein region responsible for the localization of MAP2, the serine/proline-rich (S/P) region. Our pulse-chase live-cell imaging revealed the slow but steady migration of MAP2 toward distal dendrites, which was not observed in the presence of a dynein inhibitor. By integrating these experimental parameters into a mathematical model, we demonstrated that an intermittent active transport mechanism by dynein is sufficient to explain the observed MAP2 dynamics. In addition, our experiments using a dynein inhibitor as well as dynein knockdown showed that impaired dynein function leads to mislocalization of MAP2 in the axon. Furthermore, we verified that cytoplasmic dynein binds to MAP2 through the S/P region in heterologous cells. Finally, we show that the failure of this dynein-mediated transport mechanism results in the ectopic stabilization of axonal microtubules, thereby disrupting axonal identity. Thus, we propose that the cytoplasmic dynein recruits and transports free MAP2 toward distal dendrites, thereby maintaining the precise dendritic localization of MAP2. Our findings shed light on the previously unknown mechanism behind MAP2 localization and provide a new direction for soluble protein trafficking research in the field of cell biology of neurons.

MAP2 is one of microtubule (MT) associated proteins (MAPs) and has been known to localize predominantly to the cell body and dendrites (1, 2). Among MAPs, MAP2 and Tau are probably the best-recognized and -utilized MAPs in neurobiology, owing to their usefulness as markers of neuronal dendrites and the axon, respectively. The Primary function of MAP2, like Tau, is thought to be binding to MT to stabilize them (3). Alternative splicing of *MAP2* gives rise to four isoforms, MAP2A and B (high molecular weight, HMW), and C and D (low molecular weight, LMW). Expression of these isoforms is differently regulated during neuronal development. MAP2B is expressed throughout development, and the expression level of MAP2A increases during development. In contrast, expression level of MAP2C and D decreases during development, although LMW MAP2 still exists in adulthood (4). A physiological role of somatodendritic localization of MAP2, particularly near the hillock of the axon, has recently been proposed as a facilitator of the axonal cargo sorting before entering the axon initial segment (5). Despite the utility and significance of the somatodendritic localization of MAP2, still very little is known about how the specific localization is established and maintained.

There have been attempts to understand how MAP2 localizes to soma and dendrites and not to the axon. We reckon the following three major hypotheses proposed over the years; (i) preferential binding of MAP2 to dendritic MT (6, 7), (ii) localization of *MAP2* mRNA in dendrites for local translation (8), and (iii) rapid turnover of MAP2 in the axon (7, 9). However, (i) preferential binding of MAP2 to dendritic MT should be confirmed in living neurons, (ii) localization of *MAP2* mRNA might not be a key factor because MAP2 without 3′-UTR typically required for mRNA transport still properly localizes to soma and dendrites (10), and (iii) observed “rapid turnover” of MAP2 in axon can also be explained by sorting out from axon instead of turnover. Kanai *et al.* (6) also reported that the projection domain (PD) of MAP2, which exists only in MAP2A and B, prevents MAP2 from entering the axon, resulting in the somatodendritic localization of HMW MAP2, although LMW MAP2C lacking PD still localizes to the soma and dendrites (11). Therefore, there has not been a convincing model, which can account for the highly restricted localization of MAP2, including LMW MAP2s.

In this study, we sought to identify molecular mechanisms of somatodendritic localization of MAP2. We investigated factors involved in regulating the localization of MAP2 and found that the serine/proline rich region (S/P region) of MAP2 is critical for its specific localization to the soma and dendrites. Live-cell pulse-chase imaging using a photoconvertible fluorescent protein, Dendra2, revealed that MAP2 is transported toward the tip of a dendrite, and that this process is dependent on dynein. Our data-driven mathematical model using Virtual Cell (VCell) (12) showed that a combination of diffusion and intermittent active transport is consistent with biased MAP2 migration in dendrites. Furthermore, inhibiting cytoplasmic dynein resulted in axonal leakage of endogenous MAP2, and co-IP experiment showed MAP2 binding to dynein is S/P region-dependent, suggesting that dynein transports MAP2 through the S/P region and localizes MAP2 to the somatodendritic compartment. Importantly, mislocalization of MAP2 in the axon ectopically stabilized axonal microtubules, potentially perturbing axonal transport and cytoskeletal dynamics. Based on these results, we propose a new model for the somatodendritic-specific localization of MAP2, which has not been unequivocally shown for decades.

## Results

### Important protein domain of MAP2 for the somatodendritic localization

**Figure 1:**
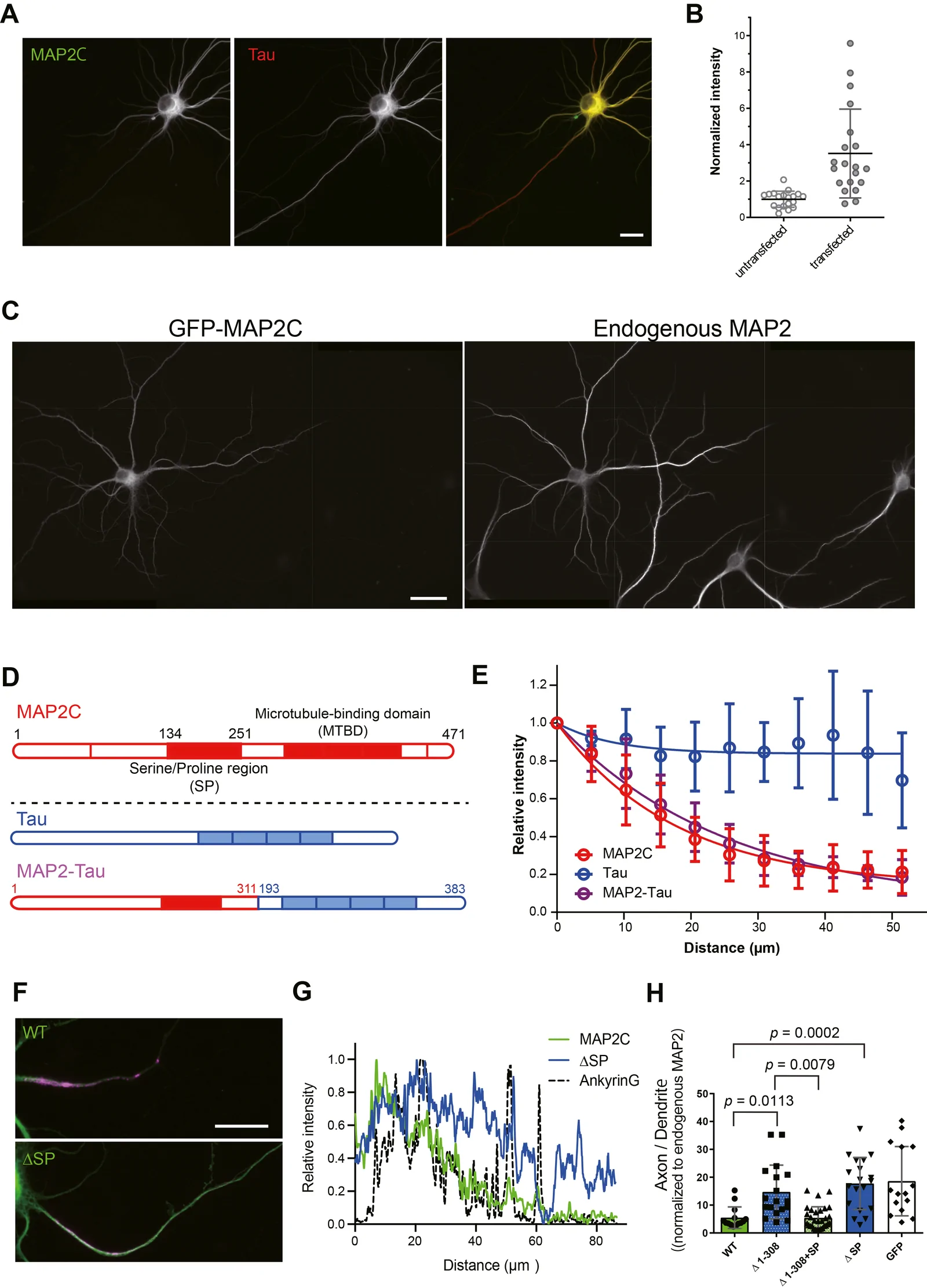
**Determinant of the somatodendritic localization of MAP2C.** A, somatodendritic localization of GFP-tagged human MAP2C expressed in cultured neurons with mCherry-tagged human Tau. Scale bar, 20 μm. *B*, estimation of expression levels of GFP-MAP2C using immunofluorescence staining with anti-pan MAP2 antibody (MAP2N), which detects both endogenous and exogenous MAP2 isoforms. Fluorescence intensities in the soma were measured and normalized to the average intensity in nontransfected neurons. Scatter plot shows mean ± SD with n = 19 (Nontransfected neurons) and 20 (transfected neurons) from two independent experiments (4 coverslips). *p* < 0.0001 (two-tailed Mann–Whitney U test). *C*, immunofluorescence labeling of untransfected and transfected neurons with GFP-MAP2C for endogenous HMW MAP2 (*right panel*). *Left panel* shows a neuron transfected with GFP-MAP2C. *D*, Schematic diagram showing the domain structure of MAP2C and chimeric proteins with Tau. *E*, relative intensity of GFP fluorescence from MAP2C, Tau, and MAP2-Tau chimera on a line drawn from the base of the axon was measured. Data are presented as mean ± SD with n = 8 (MAP2C), 6 (Tau) and 5 (MAP2-Tau) from 2 (for Tau) or three independent experiments (MAP2C and MAP2-Tau). *F*, Distribution of GFP-MAP2C and GFP-MAP2C ΔSP mutant in axon is shown with the immunolabeling of Ankyrin G (AIS marker). Scale bar, 20 μm. *G*, Line scan plots of *F*. *H*, quantification of axonal leakage of MAP2C and deletion mutants. See Experimental procedures for details. Statistical significance was assessed by Welch's ANOVA (W(4, 37.35) = 13.45, *p* < 0.0001) followed by Dunnett's T3 multiple comparisons test. Data are presented as mean ± SD with n = 14(WT), 18(Δ1-308), 30(Δ1-308+SP), 19(ΔSP), 15(GFP), from three independent experiments.

To understand how the exclusive dendritic localization of MAP2 is established and maintained, we analyzed human MAP2C tagged with AcGFP (GFP hereafter) transfected into cultured neurons at 7 days *in vitro* (DIV) and analyzed at 9 DIV. We found that GFP-MAP2C exhibited somatodendritic localization (Fig. 1*A*) despite its 3.5 ± 0.5-fold overexpression (Fig. 1*B*). It should also be noted that overexpression of GFP-MAP2C did not result in any discernable difference in endogenous MAP2 expression or localization (Fig. 1*C*). Also, we did not detect significant differences in the somatodendritic localization between MAP2C and the HMW MAP2s (Fig. S1, *A* and *B*). The somatodendritic localization of GFP-MAP2C indicates that protein domains, other than the PD found only in the HMW MAP2s, contain a signal for somatodendritic retention. To identify the critical protein domains, we generated a MAP2C-Tau chimera, in which the C-terminal half containing the microtubule-binding domain (MTBD) was swapped with that of Tau (Fig. 1*D*). Interestingly, this chimera localized like the wild-type MAP2C, as evidenced by the rapid decrease of fluorescence signals from the soma into the axon (Fig. 1*E*). These results suggest that the N-terminal half of MAP2C contains the critical domains for localization.

We therefore generated and tested a series of deletion mutants of MAP2C to identify the critical domain. MAP2C primary structure was divided into seven domains based on the amino acid composition and known functions (Fig. S1*C*), and individual domains were deleted. Among these deletion mutants, we found that the mutant lacking the S/P domain (ΔSP) exhibited substantial axonal signals even in distal axons, unlike WT MAP2C, which exhibited substantial signal reduction within the AIS (Fig. 1, *F*, *G*, and S1*D*). The mutant lacking the N-terminal region of MAP2C, including the S/P domain (Δ1-308), was also distributed to the axon (Fig. 1*H* and S1*D*). To test whether the S/P domain is responsible for the change, we put back the S/P domain to Δ1-308. This restored the somatodendritic localization (Fig. 1*H* and S1*D*), further confirming the importance of the S/P domain in MAP2C localization.

### Dendritic dynamics of MAP2 in dendrites

**Figure 2:**
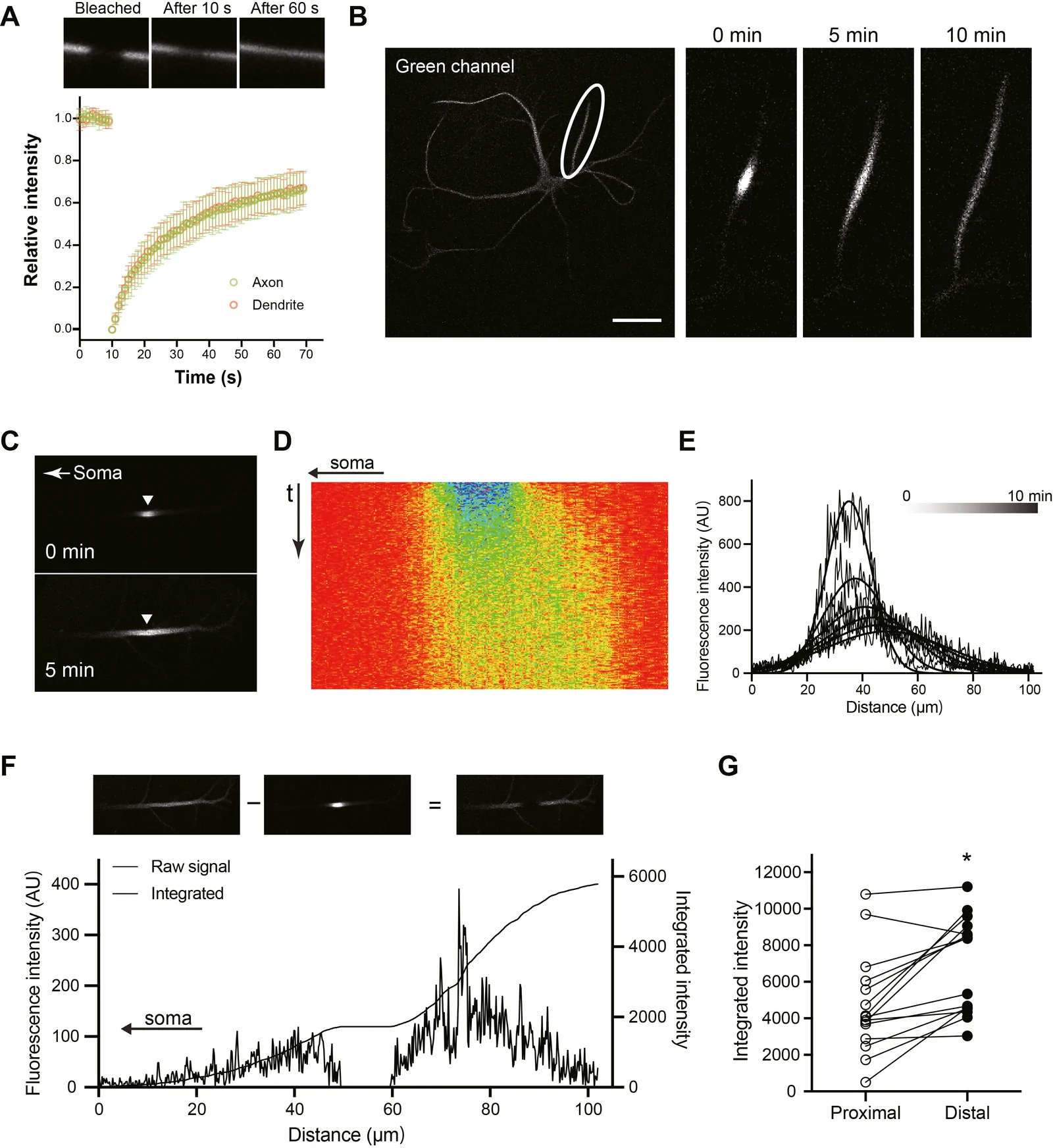
**Transport of MAP2C in dendrites.***A*, FRAP of GFP-MAP2C in dendrites and axon. Neurons are transfected with GFP-MAP2C at DIV7 and FRAP experiments were performed at DIV9. Data are presented as mean ± SD with n = 24 (axon), 22 (dendrite) from seven independent experiments. *B*, Photoconversion of Dendra2-MAP2C in a dendritic branch indicated in the *left panel* showing the overall distribution before photoconversion. *Right panels* show the time evolution of *red* fluorescence after photoconversion. Scale bar, 50 μm. *C*, migration of photoconverted Dendra2-MAP2C in a dendrite toward the dendritic tip. 0 min and 5 min indicate time after photoconversion. *D*, Kymograph of *C*. *E*, temporal changes of fluorescence distribution over line drawn over the photoconversion spot. *F*, quantification of the biased distribution of fluorescence signals at 10 min after photoconversion. Raw signal is calculated as follows, “fluorescence signal at 10 min after photoconversion” – “fluorescence signal from the first frame after photoconversion”. Representative images of 10 min, 0 min after photoconversion and after subtraction are shown. *G*, comparison of integrated fluorescence intensities in the proximal and distal sites of the photoconversion spot for WT MAP2. Each pair of connected points represents one neuron. Data were analyzed together with those in Figures 3*E* and 5*C* (WT, ΔSP, DMSO, and ciliobrevin D) by two-way ANOVA with repeated measures in one factor (proximal/distal), followed by Šídák's multiple comparisons test. The analysis revealed a significant interaction between condition and location (F(3, 41) = 6.58, *p* = 0.0010; main effect of location, F(1, 41) = 19.29, *p* < 0.0001; main effect of condition, F(3, 41) = 1.40, *p* = 0.26). For WT, the distal signal was significantly greater than the proximal signal (∗*p* < 0.0001; n = 15 from three independent experiments).

Stable MT-binding of MAP2 may help keep MAP2 in the soma and dendrites. To investigate MT-binding of MAP2C in living neurons, we performed fluorescence recovery after photobleaching (FRAP) experiments. Interestingly, the recovery in the second timescale indicated that MAP2 MT-binding in both dendrites and the axon is dynamic and that a fraction of MAP2C is in the diffusible state at all times (Fig. 2*A*). To confirm that this recovery reflects MT-dependent binding dynamics, we performed FRAP in the presence or absence of nocodazole. MT depolymerization by nocodazole further accelerated the fluorescence recovery of MAP2C (Fig. S2) compared to the untreated control, confirming that the dynamic behavior of MAP2C is MT-dependent and that MAP2C enters an almost freely diffusible state upon MT depolymerization. The dynamic MT-binding of MAP2C suggests that there must be mechanisms to keep free MAP2 in dendrites actively. Since MAP2 has been shown to bind to motor proteins in neurons (13), we hypothesized that active transport mechanisms maintain the dendritic localization of MAP2. To test this, we attempted to visualize MAP2 transport in dendrites. We prepared a construct encoding MAP2 tagged with a photoconvertible fluorescent protein, Dendra2, and performed pulse-chase imaging experiments (Fig. 2*B*). These experiments revealed that photoconverted MAP2 in a small spot in a dendrite spread slowly over time to both proximal and distal directions (Fig. 2*C*), presumably due to the cycle between diffusion and MT-binding. However, it was clear in kymographs that the redistribution of Dendra2-MAP2C was biased toward the distal direction (Fig. 2*D*), which was also revealed as the shift of line profiles (Fig. 2*E*). To quantify this, the first frame after photoconversion was subtracted from the frame at 10 min, and signals to the proximal and distal sides from the conversion spot were measured (Fig. 2*F*). In most cases, the signal was redistributed more to the distal direction than the proximal direction of the dendrite (Fig. 2*G*), indicating slow dendritic transport of MAP2C toward distal dendrites.

### Dendritic transport of MAP2 by dynein in dendrites

**Figure 3:**
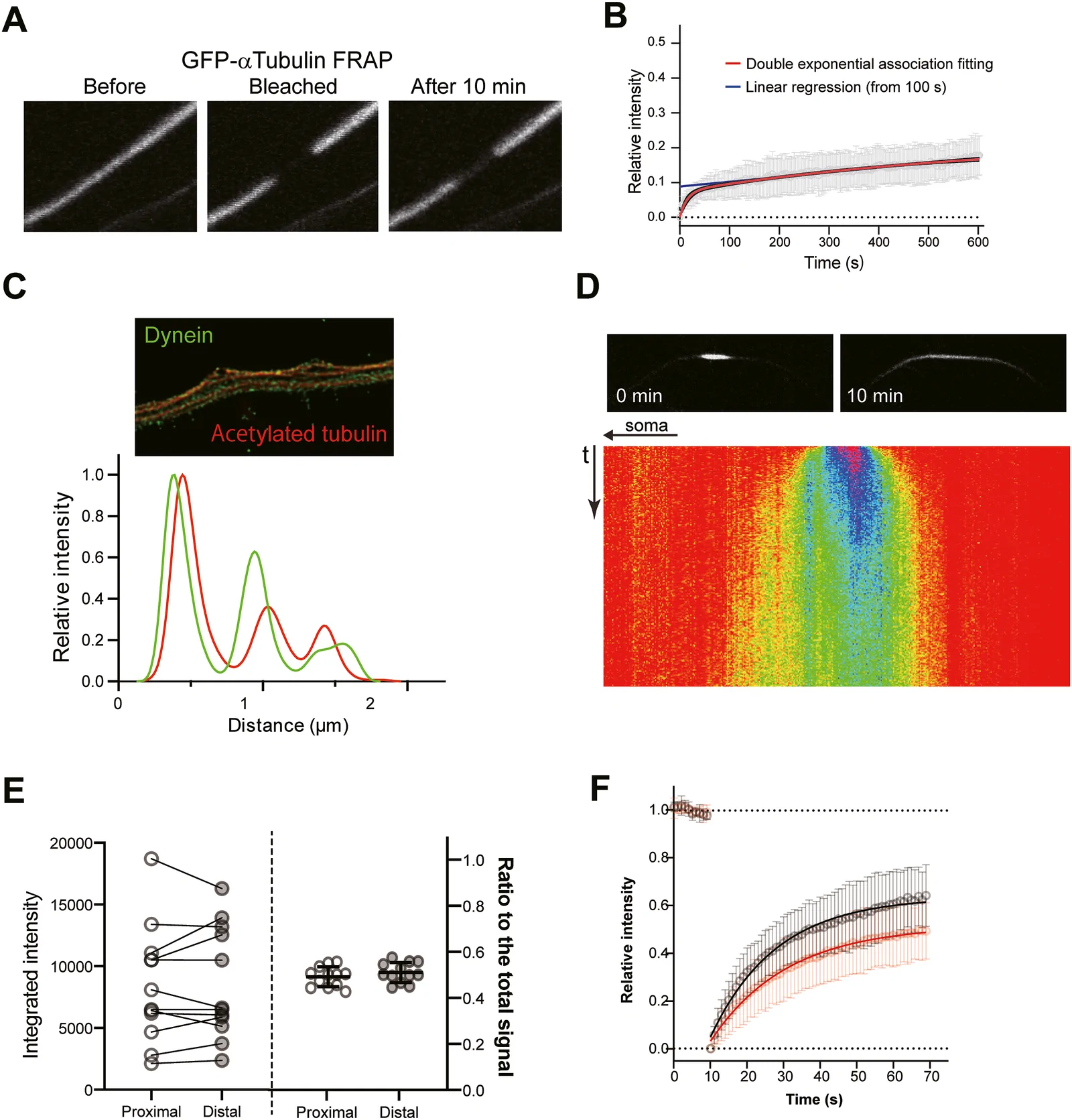
**Dendritic transport of MAP2 bydynein.***A*, representative images of FRAP experiments where GFP-α Tubulin in dendrites at 10 DIV neurons were analyzed. *B*, Group data of FRAP on GFP-α Tubulin. Data were fitted with a double exponential association curve and a linear function to estimate fractions of MTs. Data are presented as mean ± SD with n = 12 from two independent experiments. *C*, STED imaging of immunolabeling of DHC1 and acetylated MTs in dendrites (*inset*). Line scans of fluorescence labeling are shown in the graph. *D*, images of Dendra2-MAP2C immediately after photoconversion and 10 min later (*top panels*) and corresponding kymograph (*bottom*) in neurons treated with 50 μM ciliobrevin D for 6 h. *E*, Comparison of integrated fluorescence intensities in the proximal and distal sites of the photoconversion spot (*left*) and ratios of intensities over the total signal intensities (*right*) in the presence of ciliobrevin D. For the *left panel*, statistics are from the same two-way ANOVA as in Figure 2*G* (Šídák's multiple comparisons test; ciliobrevin D, *p* = 0.997, n = 12). For the *right panel*, distal ratios were compared against a theoretical value of 0.5 (equal proximal/distal distribution) by the Wilcoxon signed-rank test; the ratio for ciliobrevin D was not significantly different from 0.5 (*p* = 0.62, n = 12). The DMSO vehicle control retained the distal bias in both analyses (two-way ANOVA, *p* = 0.0014; Wilcoxon signed-rank test, *p* = 0.0039, n = 9), indicating that the loss of biased transport reflects dynein inhibition rather than the treatment procedure. Data are presented as mean ± SD; n = 12 (ciliobrevin D) and 9 (DMSO) from three independent experiments. *F*, FRAP of GFP-MAP2C in DMSO-treated control neurons and ciliobrevin D-treated neurons. The two data sets were fitted with exponential curves with different slopes but could also be fitted with a single curve with no statistical differences. Data are presented as mean ± SD with n = 9 (DMSO) and 9 (ciliobrevin D) from three independent experiments.

**Figure 4:**
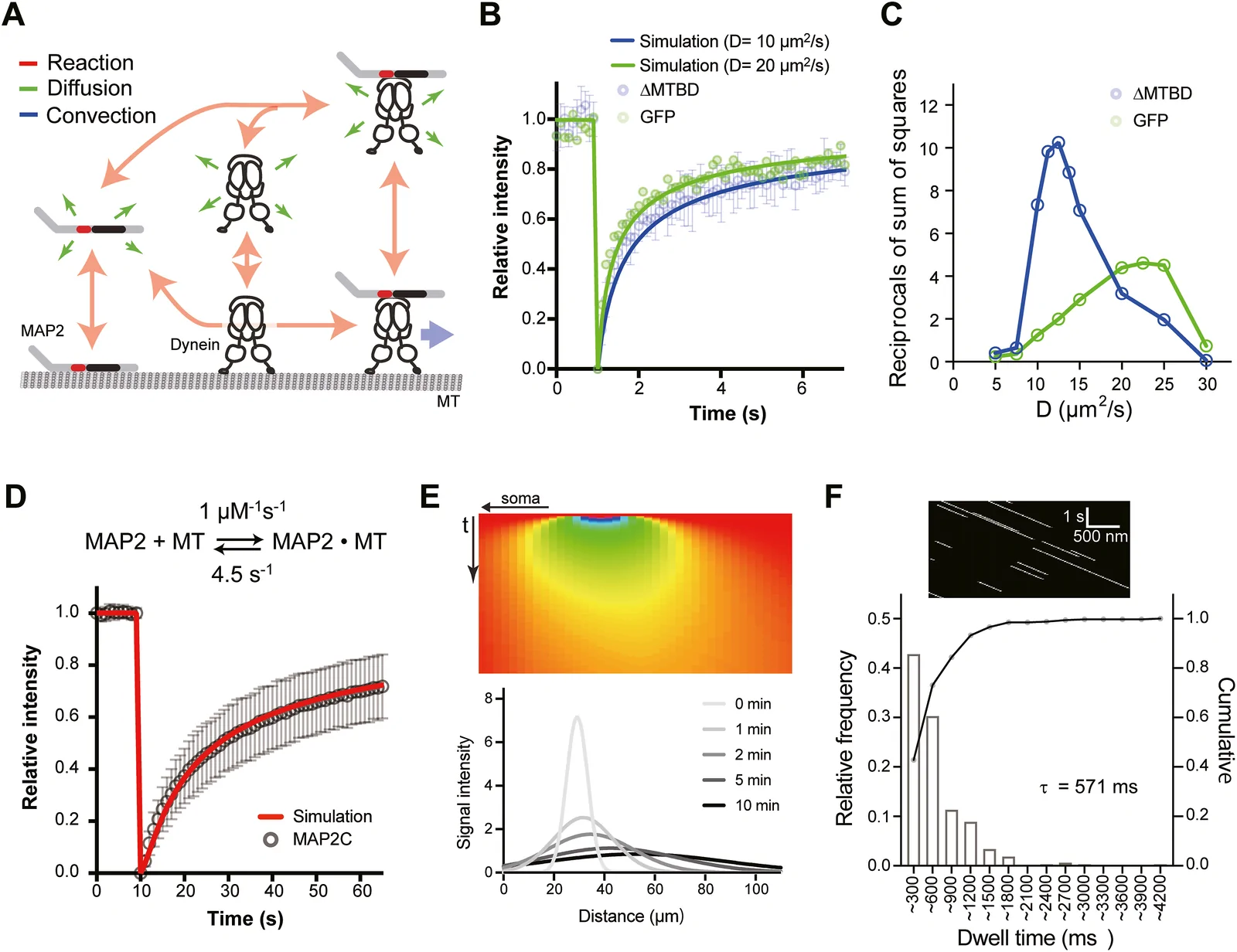
**Modeling dendritic transport of MAP2.***A*, scheme of the transport model. *B*, Estimation of diffusion coefficient from FRAP data of GFP and Tau lacking MTBD (ΔMTBD) by simulating the FRAP experiment. Data are presented as mean ± SD, n = 6 from two independent experiments. *Blue* and *green* curves indicate the simulation data with diffusion coefficient 10 or 20 μm^2^/s, respectively. *C*, fitting of simulation data on FRAP data with varying diffusion coefficient. Reciprocals of sum of squares are plotted against diffusion coefficient. *D*, estimation of MT-binding kinetics of MAP2C from FRAP data (open circles, mean ± SD, n = 10 from three independent experiments). The *red* curve indicates the simulation data with the reaction kinetics shown in the reaction equation. *E*, The *upper panel* shows virtual kymograph of the converted MAP2 and the *lower panel* shows line scan analysis from model simulation at indicated time point after conversion. *F*, stochastic simulation of the model. The image shows a kymograph of the MAP2-dynein-MT transport complex, which moves unidirectionally. The graph is the histogram of MAP2 dwell time in the transport complex. The characteristic dwell time (time constant, τ) was 571 ms, indicating that individual transport events are short-lived.

The slow migration of MAP2C indicates either a motor-based mechanism or an MT-polymerization-related mechanism. To test if we can observe substantial MT dynamics in dendrites of 10 DIV neurons, we performed FRAP experiments using neurons transiently expressing GFP-tagged α-tubulin. The data showed that tubulin is highly immobile in dendrites in the relatively mature neurons (Fig. 3*A*). From the data, we estimated that less than 10% of tubulin molecules are used for active MT polymerization (Fig. 3*B*) and speculate that the observed movement of photoconverted MAP2C seems not to be due to growing MTs. This was also confirmed using numerical simulation (see Figs. 4 and S3). We therefore turned to the motor-based mechanism. What could be the motor protein for the anterograde transport of MAP2 in dendrites? Unlike in the axon, MTs in dendrites have been shown to be mixed in their orientations (14). If this is the case, motors would not be able to transport MAP2 more preferentially to distal dendrites. However, recent studies suggest that acetylated MTs are oriented uniformly with their minus ends toward the distal end in dendrites (15). Since cytoplasmic dynein transports cargo toward the minus end of MT and binds preferably to acetylated MTs like kinesins (16, 17), dynein may utilize acetylated MTs as a preferred track for the dendritic transport of MAP2. Supporting this possibility, our super-resolution microscopy analysis revealed that dendritic cytoplasmic dynein heavy chain 1 (DHC1) labeling along with acetylated MTs (Fig. 3*C*). These observations led us to hypothesize that dynein is the primary motor responsible for MAP2 transport. To test this, neurons expressing Dendra2-MAP2C were treated with 50 μM ciliobrevin D, a specific inhibitor of cytoplasmic dynein (18), for 6 h, and subjected to pulse-chase imaging in the presence of the inhibitor. Intriguingly, the inhibitor disrupted the anterograde transport of MAP2C in dendrites (Fig. 3*D*), such that there was no obvious biased redistribution of converted MAP2C (Fig. 3*E*). FRAP analysis showed that 24-h ciliobrevin D treatment caused a slight but significant decrease in the recovery of MAP2C fluorescence (Fig. 3*F*).

### Data-driven model of MAP2 diffusion, binding, and transport

To further evaluate whether the biased and slow redistribution observed in the photoconversion experiments could arise from a combination of diffusion, binding and motor-driven transport, we sought to build a mathematical model of MAP2C transport using experimentally or literature-derived parameters (Fig. 4*A*). First, we made an estimation of MAP2C diffusion based on FRAP data. We have extensively used FRAP to analyze MT-binding of Tau, a sibling MAP, and previously reported that Tau lacking the microtubule-binding domain (ΔMTBD) is freely diffusible (5). We simulated the FRAP experiment with GFP and ΔMTBD in a thin cylindrical neurite (in this case axon) using the diffusion equation with a varying diffusion coefficient under the VCell environment (Fig. 4*B*). We estimated best-fit values by plotting the reciprocals of the sum of squares between the data and simulation results and found 22.5 μm^2^/s for GFP and 12.5 μm^2^/s for ΔMTBD as respective best-fit values (Fig. 4*C*). Since wild-type MAP2C is larger than the mutant Tau, we chose 10 μm^2^/s for Dendra2-MAP2C and GFP-MAP2C. These estimates were made based on the assumption that the FRAP procedure results in the full conversion of GFP to a permanently dark state, such that the recovery solely reflects molecular motions. However, it has been reported that GFP variants can be switched to a reversible dark state (19, 20, 21). We performed FRAP after fixing the cells and observed 5% recovery within 5 s, which we assumed to reflect a fraction of GFP in the reversible dark state. We then performed FRAP simulation, in which 5% of GFP goes into the reversible dark state. Fortunately, this did not affect the estimate, presumably due to rapid diffusion of these species out of the bleached region.

Next, we estimated the concentration of MAP2 in dendrites. The concentration of endogenous MAP2 was estimated from immunoblot quantification of MAP2 in homogenates of adult mouse cortices against purified mouse brain HMW MAP2. Our measurement indicated 1.85 nmol/g wet tissue, which gave about 5.6 μM with an assumption that somata and dendrites occupy one-third of the total volume. We employed 5 μM for endogenous MAP2 and 25 μM for GFP-MAP2C because we used neurons expressing 3–5-fold higher levels of exogenous MAP2C than endogenous MAP2, based on the results in Figure 1*B*, in most of the experiments. We also estimated the concentration of MTs. Based on the structure, there would be about 3300 tubulin molecules (1650 α/β heterodimers) per 1 μm length of MT. It has been reported that there are on average, about 70 MT/μm^2^ in a dendrite (14, 22, 23). Therefore, in 1 μm^3^ there could be 230,000 tubulin molecules, which translates to about 400 μM. Assuming that 2–3 tubulin heterodimers provide a MAP2-binding site with three MT-binding repeats (24), we estimated 75 μM potential MAP2-binding sites (MTs in the simulation). In an immunoblot quantification, we obtained about 80 nmol/g wet tissue of α-tubulin. From this, we estimated 320 μM total (α and β) tubulin and 55 μM MT, if neurons occupy 50% of the tissue volume, and if 85% of tubulin contributes contribute to MTs (25). In the following simulations, we mainly utilized the 75 μM estimate derived from the aforementioned deduction but obtained comparable results with 40–55 μM MT with minor adjustments in the binding affinity between MAP2 and MTs (see below).

We then simulated FRAP data of wild-type GFP-MAP2C using the reaction-diffusion equation with varying binding parameters. The data were fitted well with *K*_D_ = 4.5 μM, when *k*_on_ was set above 0.1 μM^−1^s^−1^ (Fig. 4*D*). We mainly used *k*_on_ of 1 μM^−1^s^−1^ for the following simulations, as there were no discernible differences between 0.1 and 1 μM^−1^s^−1^. With 40 μM MT, the data were fitted well with *K*_D_ = 1 μM. The diffusion coefficient and MT-binding parameters of dynein were determined based on the literature. MAP2-dynein affinity was determined based on the affinity for linker peptides (26, 27). The concentration of dynein was set at 5 μM arbitrarily. Parameters used for the model are summarized in Table 1.

**Table 1:** Condition for the Simulation

Diffusion coefficients	
Free MAP2	10 μm^2^/s
Dynein-bound MAP2	1 μm^2^/s
MT-bound species	0.001 μm^2^/s
Motor speed	1 μm/s
Binding affinity (K_D_)	
MAP2-MT	4.5 μM^a^
MAP2-dynein	10 μM^b^
Dynein-MT	2 μM^c^
Initial concentrations	
Endogenous MAP2	5 μM (0 in axon)
Dendra2-MAP2 (green)	25 μM (0 in axon)
Dendra2-MAP2 (red)	0 μM
Dynein	5 μM
MT (binding sites)	75 μM

a The value was estimated based on the initial putative *k*_on_ = 1 μM^−1^s^−1^. Note that *K*_D_ = 3.3 μM with *k*_on_ = 0.09 μM^−1^s^−1^ provided comparable fit to the FRAP data and worked similarly on simulation.

b *K*_D_ was estimated from literatures for linker-dynein binding (26, 27) and from the Co-IP experiment. *K*_on_ used for this estimation was 0.1 μM^−1^s^−1^.

c The *K*_D_ was based on previous reports (61, 62, 63, 64) and *K*_off_ from dynein dwell time on MTs (64).

To simulate the dendritic photoconversion imaging, a 200 μm long cylinder with 3 μm diameter was built in the VCell. To implement active transport, reaction-diffusion-convection (advection) equations were applied to dynein species bound to both MT and MAP2. To avoid redistribution of MTs, which are expected to be virtually immobile, the concentration of MT was made constant in each unit volume. The velocity of convection, *i.e.*, the speed of motor, was set to 1 μm/s. Photoconversion took place in the middle of the cylinder. Virtual kymograph of the converted species (red MAP2) showed a gradual spreading of the species due to diffusion and drifting of the center of distribution because of the transport (Fig. 4*E* upper panel). This was similar to the kymograph from imaging experiments. The line scan profiles were also similar to the data (Fig. 4*E* lower panel). We also tested whether a fraction of polymerizing MTs, determined by the FRAP experiment (Fig. 3*A*), is sufficient to account for the drift. We set 10% of MTs to move toward distal dendrites at the net speed of 0.1 μm/s with MAP2, considering the reported speed of MT polymerization at about 0.3 μm/s (Fig. S3). This did not exhibit the drift speed we observed experimentally, confirming that the motor-dependent mechanism is more appropriate.

We also tested the motor-dependent model in a bottom-up particle-based stochastic simulation using Smoldyn based on the Smoluchowski model of diffusion influenced systems (28) in the VCell environment with the same parameters. This enabled us to estimate how individual MAP2 molecules behave with the given condition. The model estimated that about 87% of MAP2 is bound to MTs, 8.4% free, and 4.2% being transported (bound to motor and MT) at a given point in time. With the *k*_on_ and *k*_off_ that we used, MT-binding was transient with median and τ of dwell time at 140 and 239 ms, respectively, which is comparable to the predicted residence time (1/*k*_off_), 222 ms. MAP2 in the transport state was also short-lived, such that the dwell time in the transport state was less than 1 s in 90% of the cases (Fig. 4*F*). These results therefore demonstrate that our transport model, built on experimental parameters, successfully captures the slow and biased migration of MAP2C observed in neurons, supporting the idea that an intermittent active transport mechanism can account for its dendritic dynamics.

### Dendritic transport of MAP2 is impaired by the ΔSP mutant

**Figure 5:**
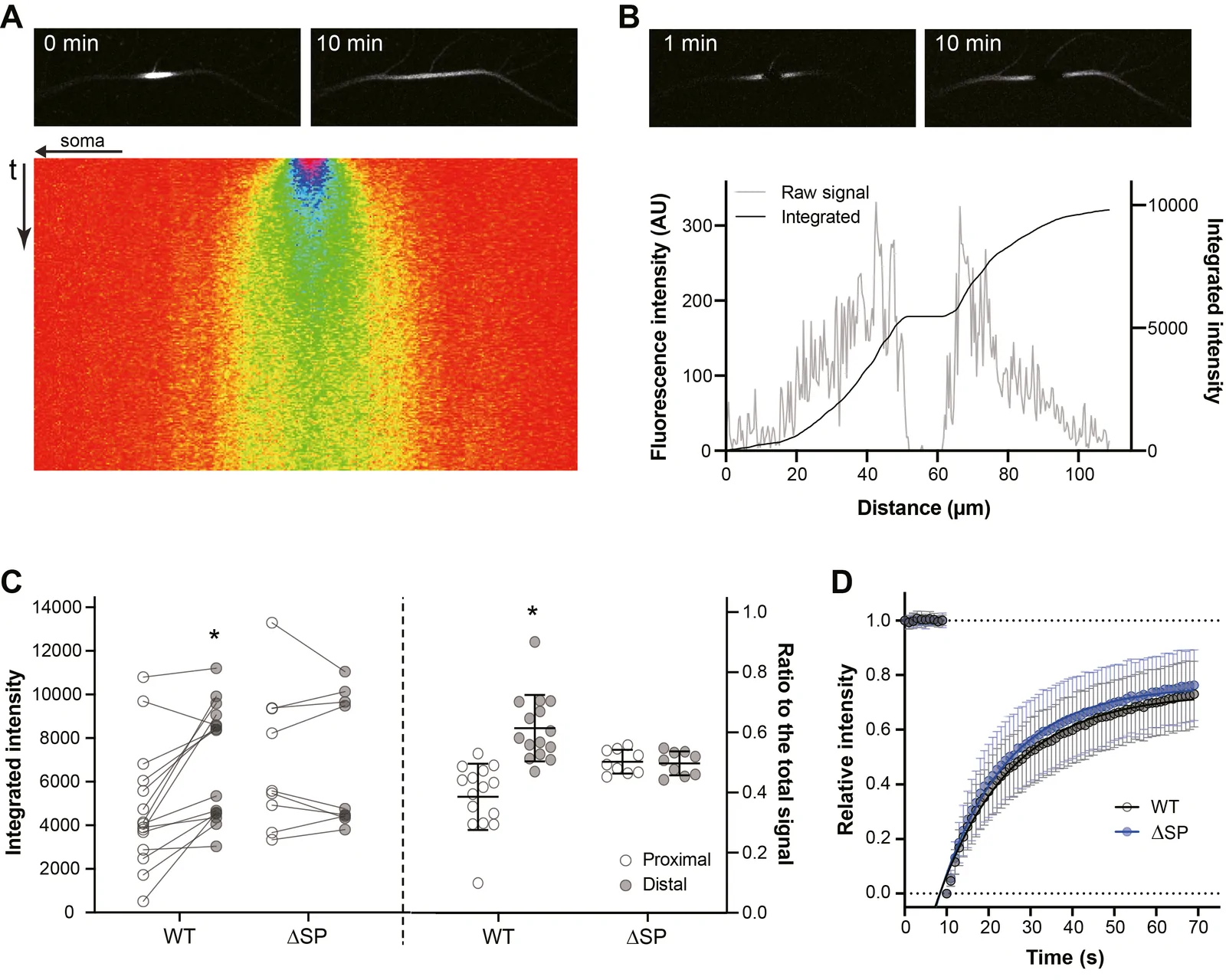
**S/P region-dependent transport of MAP2 in dendrites.***A*, representative images of Dendra2-MAP2C ΔSP immediately after photoconversion and 10 min later (*top panels*) and corresponding kymograph (*bottom*). *B*, the first frame after photoconversion was subtracted from 1 min and 10 min after photoconversion and representative subtracted images of 1 min and 10 min after photoconversion are displayed (*top panels*). Corresponding fluorescence signals at 10 min after photoconversion (*bottom*). *C*, comparison of integrated fluorescence intensities in the proximal and distal sites of the photoconversion spot for ΔSP (*left*) and ratios of intensities over the total signal intensities (*right*). Note that the wild-type (WT) data in the left panel are the same data shown in Figure 2*G*. For the *left panel*, statistics are from the same two-way ANOVA as in Figure 2*G* (Šídák's multiple comparisons test; ΔSP, *p* = 0.999, n = 9 from three independent cultures). For the *right panel*, distal ratios were compared against a theoretical value of 0.5 by the Wilcoxon signed-rank test; the ratio for ΔSP was not significantly different from 0.5 (*p* = 0.82, n = 9 from three independent cultures), in contrast to WT (*p* = 0.0004), which showed a significant distal bias. Data are presented as mean ± SD. *D*, FRAP of GFP-MAP2C WT and ΔSP in dendrites. The two data sets were fitted with exponential curves with different slopes but could also be fitted with a single curve with no statistical differences. Data are presented as mean ± SD with n = 10 (WT) and 8 (ΔSP) from at least three independent experiments.

We then investigated whether this dendritic transport is altered by S/P-deletion, which resulted in mislocalization to the axon (Figs. 1, *F*–*H* and S1*D*). Kymograph analysis after photoconversion in dendrites showed that the ΔSP mutant evenly spread from the conversion spot to both proximal and distal directions (Fig. 5*A*). Quantitative analysis confirmed this (Fig. 5*B*). The group data also showed that ΔSP no longer exhibited the biased redistribution observed with WT (Fig. 5*C*). One plausible explanation for this is that more ΔSP is in the free and diffusible fraction than WT. FRAP analysis in dendrites suggests that this is not the case. ΔSP and WT were virtually identical in the rate and magnitude of fluorescence recovery (Fig. 5*D*). Taken together, these results suggest that both dendritic transport and the somatodendritic localization depend on the S/P domain of MAP2 and dynein.

### Cytoplasmic dynein-dependent maintenance of MAP2 localization

**Figure 6:**
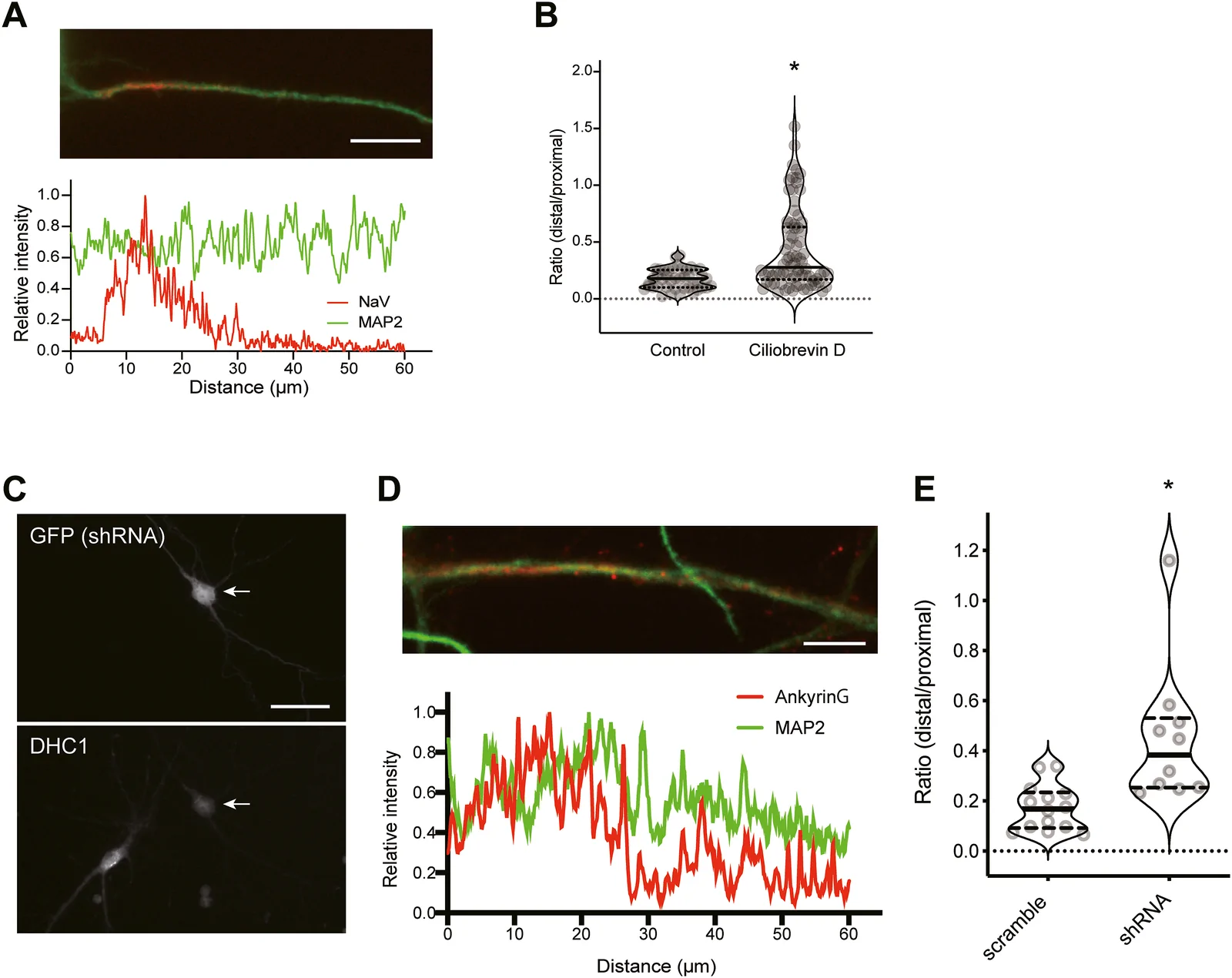
**Mislocalization of endogenous MAP2 upondynein depletion.***A*, representative image of endogenous MAP2 in neurons treated with ciliobrevin D for 48 h, shown by immunofluorescence co-labeling of MAP2 and pan-sodium channels, NaV (*top*). The bottom panel shows the signal intensities of MAP2 and NaV from top panel over a line drawn from the base of the axon. *B*, ratios of MAP2 signal intensities in the distal axon (distal to the AIS) *versus* the proximal axon in neurons treated with DMSO (Control) or ciliobrevin D. Each data point and violin plots are shown. n = 77 (DMSO) and 88 (ciliobrevin D) from at least three independent experiments. ∗*p* < 0.0001 (two-tailed Mann–Whitney U test). *C*, neurons transfected with a plasmid containing shRNA against DHC1 and GFP (*top panel*). Neurons stained with anti-DHC1 antibody. Scale bar, 50 μm. *Arrow* indicates transfected neuron. *D*, representative image of endogenous MAP2 and Ankyrin G in neurons transfected with dynein shRNA. Scale bar, 20 μm. *E*, ratios of distal axonal signals over proximal signals of MAP2 immunolabeling in neurons transfected with scrambled or dynein shRNA. Each data point and violin plots are shown. ∗*p* = 0.0002 with two-tailed Mann-Whitney test. n = 14 (scramble), 10 (sh-DHC1) from three independent experiments.

We next asked whether the loss of dynein function itself leads to the mislocalization of endogenous MAP2. To test this, neurons at 14 DIV were treated with 50 μM ciliobrevin D for either 24 h or 48 h and analyzed for endogenous MAP2 localization. Since we obtained more obvious results with the 48 h treatment, we focused on this time point. It should be noted that neurons were alive and exhibited normal morphology after the treatment (Fig. S4, *A* and *B*). As shown in Figure 6*A*, some of treated cells exhibited clear axonal MAP2 immunostaining in the axon, which was not observed in the DMSO control (Fig. S4*C*). The line scan analyses also showed the axonal distribution of MAP2 after ciliobrevin D treatment (Fig. 6*A*). We then quantified the ratio of MAP2 in the distal axon over MAP2 signal in the proximal axon (hillock) and found that the ratio is significantly greater than control (Fig. 6*B*). To further substantiate the role of dynein, we also tested cytoplasmic dynein knockdown. We used a shRNA for rat cytoplasmic DHC1, which has been reported to reduce it in cultured rat Sertoli cells (29) (Fig. 6*C*). As shown in Figures. 6*C*, and S5, *A*, *B*, GFP-positive shRNA-transfected neurons showed significantly reduced DHC1 signal compared to untransfected neurons. Dynein knockdown did result in leakage of MAP2 into the axon similar to that observed with the pharmacological inhibition (Figs. 6, *D*, *E* and S5*C*). These results suggest that the cytoplasmic dynein-dependent transport of MAP2 toward distal dendrites is important for the maintenance of the somatodendritic localization of MAP2. Loss of the ankyrinG-based AIS has been shown to disrupt the maintenance of neuronal polarity and to allow the axon to acquire molecular features of dendrites, such as axonal MAP2 (30) and postsynaptic proteins (31). To address the possibility that disruption of the AIS-based diffusion barrier could contribute to MAP2 mislocalization, we examined AnkG immunostaining (Fig. S6). The AIS remained intact in ΔSP-expressing neurons, with AnkG signal intensity comparable to that of WT-expressing (Fig. S6*A*) or untransfected neighboring neurons in the same imaging field (Fig. S6*B*). Although a modest reduction in AnkG signal was observed following prolonged dynein inhibition by ciliobrevin D (Fig. S6, *C* and *D*) or DHC1 knockdown (Fig. S6, *E* and *F*), likely reflecting the reported role of dynein in maintaining axonal microtubule organization (32), the intact AIS in ΔSP-expressing neurons indicates that AIS disruption is not required for MAP2 mislocalization.

### Binding of MAP2 to dynein complex *in vitro* and *in vivo*

**Figure 7:**
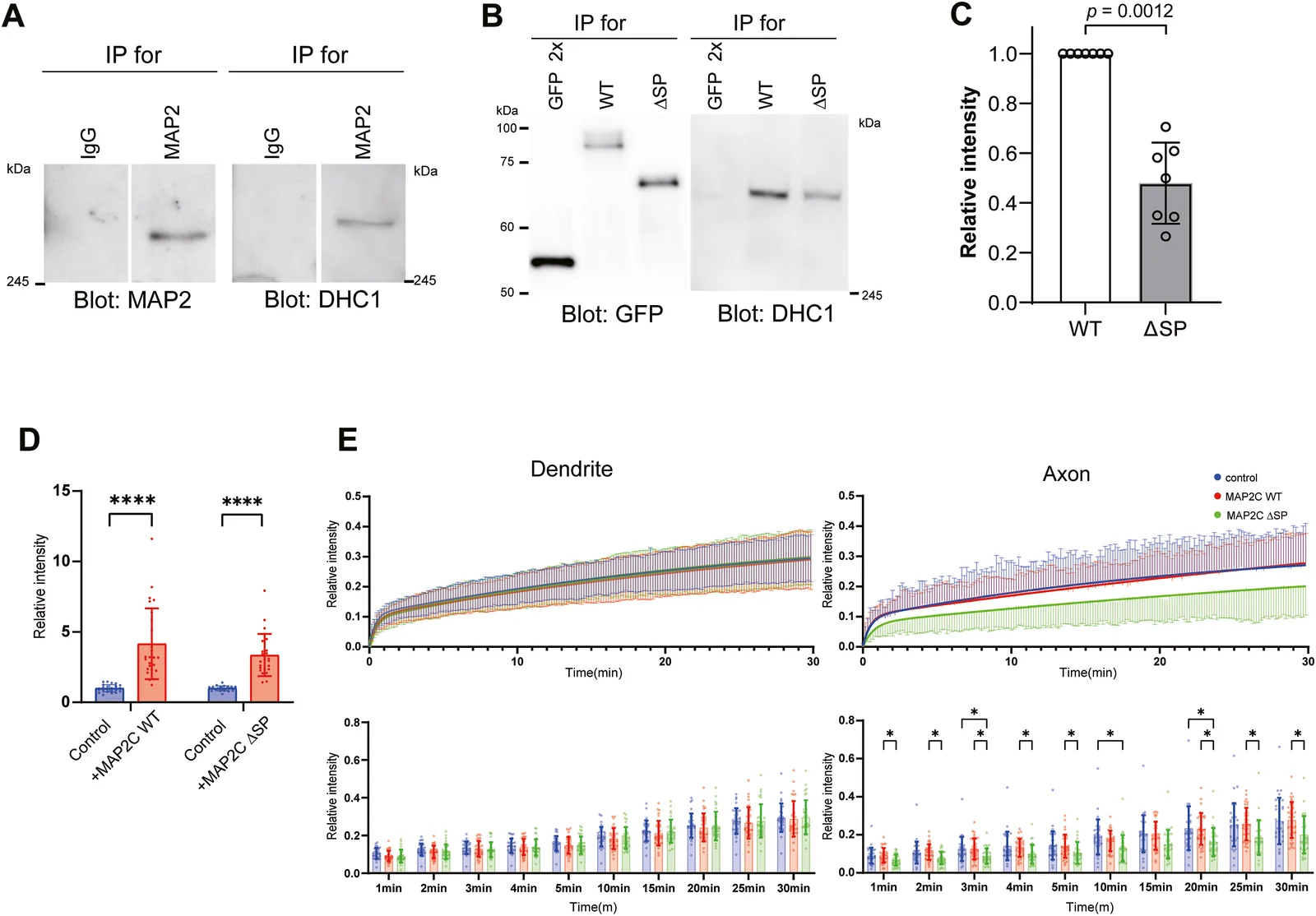
**S/P region-dependent MAP2-dynein binding and consequence of mislocalized MAP2.***A*, immunoprecipitation of MAP2 and co-immunoprecipitation of DHC1 from mouse cerebral lysates. Rabbit IgG was used as negative control. *B*, Co-immunoprecipitation of DHC1 from HEK293 cells expressing DHC1 and WT GFP-MAP2C, GFP-MAP2C ΔSP, or tandem GFP (GFP 2x with His-tag) using an antibody against His. *C*, quantification of signal intensities of co-immunoprecipitated DHC1 with WT and ΔSP MAP2C. The amount of co-immunoprecipitated DHC1 was normalized to immunoprecipitated GFP-MAP2C and expressed relative to the WT condition (set to 1). ΔSP showed significantly reduced DHC1 binding (geometric mean = 0.45, 95% CI 0.32–0.63, *p* = 0.0012, one-sample ratio *t* test, n = 7 independent experiments). Data are presented as mean ± SD; the geometric mean and 95% CI are given above. *D*, quantification of overexpressed MAP2C WT or ΔSP. Lentivirally transduced neurons were immunostained for MAP2, and fluorescence intensity was quantified and expressed relative to the control mean (each experiment normalized to its own control). Data are mean ± SD; n = 25 (control) and 26 (WT), and n = 26 (control) and 24 (ΔSP) cells from two independent experiments. ∗∗∗∗*p* < 0.0001, two-tailed Mann–Whitney U test. *E*, effect of MAP2C WT and ΔSP on the dynamics of endogenous β-tubulin. FRAP experiments were performed using 14 to 15 DIV neurons in which EGFP was knocked in at the C-terminus of β-tubulin. mCherry, mCherry-MAP2C WT or ΔSP was expressed *via* lentiviral transduction. *Upper left panel* shows results of FRAP at dendrites (20 μm from soma) and lower *left panel* shows fluorescence recovery of β-tubulin at indicated time points. *Upper right panel* shows results of FRAP at axon (80 μm from soma) and *lower right panel* shows fluorescence recovery of β-tubulin at indicated time points. Images were acquired every 10 s for 180 frames following photobleaching. Data are presented as mean ± SD. For dendrites, n = 33 (control), 34 (WT) and 34 (ΔSP) from 16 independent experiments. For FRAP at axon, n = 34 (control), 36 (WT) and 31 (ΔSP) from 15, 16, and 14 independent experiments, respectively. *Blue*, *red* and *green* indicate mCherry, mCherry-MAP2C WT and mCherry-MAP2C ΔSP, respectively. ∗ indicates *p* < 0.05 using a mixed-effects model (REML) with Geisser–Greenhouse correction (condition: *F*(1.928, 607.3) = 45.73, *p* < 0.0001; interaction: *F*(17.35, 607.3) = 0.7367, *p* = 0.7688), followed by Tukey's multiple comparisons test.

We investigated whether MAP2 binds to the dynein transport complex *in vitro* and *in vivo* and whether the binding depends on the S/P domain. We first examined their binding *in vivo* in the mouse brain. MAP2 was immunoprecipitated from the brain lysate, and the precipitate was analyzed for MAP2 and cytoplasmic DHC1. Long isoforms of MAP2 were immunoprecipitated, and DHC1 was also readily detected in the sample (Figs. 7*A* and S7, *A*, *B*), indicating that MAP2 and DHC1 can form a protein complex in the adult mouse brain.

We next investigated if the binding of DHC1 depends on the S/P domain of MAP2. First, to reconstitute their binding, wild-type or ΔSP MAP2C tagged with GFP and DHC1 were expressed in HEK293 cells, which express endogenous DHC1 at low levels as well (data not shown), and cell lysates were prepared as described in the Materials and Methods. As a negative control, we expressed tandem GFP (GFP ×2 with His-tag) and DHC1 in HEK293 cells and prepared lysate. These lysates were incubated with anti-His tag antibody for GFP-MAP2C, which has the His-tag between GFP and MAP2C. This resulted in the co-immunoprecipitation of MAP2C and DHC1 and demonstrated their binding *in vitro* (Fig. 7*B*). However, ΔSP exhibited decreased levels of co-immunoprecipitated DHC1 (Figs. 7, *B*, *C* and S7, *C*, *D*). These results suggest that the S/P region is critical for DHC1 binding, MAP2 transport, and localization of MAP2, thereby supporting our hypothesis.

### Functional impact of MAP2C ΔSP

Our findings suggest that dynein actively maintains MAP2 within the somatodendritic compartment, and that impairment of this mechanism leads to the mislocalization of MAP2 to the axon. To elucidate the functional consequences of this axonal MAP2 invasion, we next asked whether leaked MAP2C affects microtubule dynamics in the axon. For this purpose, we used CRISPR/Cas9 to knock in EGFP at the C-terminus of endogenous β-tubulin and overexpressed mCherry-tagged MAP2C WT or ΔSP *via* lentiviral infection. MAP2C WT/ΔSP transduced neurons showed 4.2- and 3.4-fold higher MAP2 staining compared to untransduced control neurons, respectively (Fig. 7*D*). In dendrites, FRAP analysis showed no significant difference in β-tubulin recovery among control, WT and ΔSP groups (Fig. 7*E* left). In contrast, MAP2C ΔSP significantly stabilized microtubules in the axon where MAP2C is normally excluded (Fig. 7*E* right). This result indicates that dynein- and S/P domain-dependent exclusion of MAP2C is critical for maintaining the physiological microtubule dynamics in the axon.

## Discussion

### Dynein-based transport mechanism

Our experimental results showed that transport and localization of MAP2 are inhibited by S/P deletion and dynein inhibitor or knockdown. Notably, we found that dynein coimmunoprecipitates with MAP2 in an S/P region-dependent manner, suggesting that the S/P region mediates the association between MAP2 and the dynein complex, possibly through an unidentified adaptor protein. Therefore, we hypothesize that dynein complex captures free MAP2 and maintains MAP2 in dendrites *via* its transport activity. Consistent with this model, Yu *et al.* reported that MAP2 staining in dendrites becomes indistinguishable from the axon when dendrites lose minus-end-out MTs (33). The transport of MAP2 we observed was biased toward dendrites, with an apparent velocity of ∼0.03 μm/s, which is within the reported velocity range of the dendritic transport of RNA (0.005–0.1 μm/s) (34, 35, 36).

One mechanistic question that arises from our findings is whether dynein transports MAP2 directly or indirectly. Konzack *et al.* demonstrated that Tau is transported in axons by "piggybacking" on short microtubule fragments propelled by molecular motors (37), providing a precedent for MAP2 transport by a similar mechanism. Our data show that the S/P region is essential for this process, as its deletion abolishes transport despite the intact microtubule-binding domain. This is consistent with two potential models: one in which dynein transports free MAP2 directly as cargo *via* its S/P region, and another in which dynein recognizes MAP2 bound to short microtubule fragments through the S/P region, transporting the MAP2-microtubule complex as a unit. In both models, the S/P region is central to dynein-mediated transport, distinguishing this mechanism from passive piggybacking that would be independent of the S/P region.

Crucially, we identified the functional consequence of MAP2C mislocalization: the ectopic stabilization of microtubules in the axon. This suggests that the dynein-dependent transport of MAP2 serves as a protective mechanism to prevent aberrant cytoskeletal stiffening in the axon, which could otherwise interfere with axonal transport or organelle trafficking. One aspect we have not been able to demonstrate experimentally is the retrograde transport of MAP2 in the axon, due to insufficient levels of Dendra2-MAP2 present in the axon to perform the photoconversion experiments.

Dynein, which moves toward minus-end of MTs, is reported to transport cargoes both anterogradely (38, 39) and retrogradely (40, 41) in dendrites where orientation of MTs is considered to be mixed. This raises a critical question; how can dynein transport MAP2 anterogradely into dendrites? Tas *et al.* reported that plus-end-out and minus-end-out MTs are bundled and separated in dendrites, promoting persistent moving in a certain direction because of reduced potential switching to oppositely oriented MTs (15). They also showed that acetylated and tyrosinated MTs are separated, and that acetylated MT fraction contains more minus-end-out MTs than tyrosinated MTs (66% vs 34%). Katrukha *et al.* reported using super-resolution light microscopy that in dendrites 72 ± 6% of microtubules are acetylated and 26 ± 8% are tyrosinated (23). Therefore, in dendrites, it seems that ∼50% of microtubules are oriented with minus-end out (72% × 66% = ∼48%), which is consistent with the finding that ∼50% of microtubules are oriented with minus-end out throughout the dendrite (15). Furthermore, Kapitein *et al.* demonstrated using quantitative modeling that dynein can transport cargo into dendrites where orientation of microtubules is 50% minus-end out, without considering findings that MTs are bundled and separated (38). Therefore, it seems to be possible that dynein transports MAP2 anterogradely into dendrites with a mechanism with which dynein/MAP2 complex selectively or preferentially binds to minus-end-out microtubules, or *via* its moving back and forth that is biased anterogradely. Regardless of detailed mechanism of how dynein/MAP2 moves on minus-end-out microtubules, our data suggest that dynein transports MAP2 into dendrites.

### Dynamic MT-binding

Our FRAP data and stochastic simulation suggest that MT-binding of MAP2 in living neurons is quite dynamic, with an estimated *K*_D_ of 1–3 μM (depending on the estimated level of MTs). This was surprising because MAP2 binds to MTs quite stably in *in vitro* binding assays (42). Overexpressed GFP-MAP2C might saturate MTs, thereby increasing the fraction of free GFP-MAP2C. Our empirical and experimental estimation shows that there are 60–100 times more tubulin molecules than MAP2 in the cytoplasm. Therefore, MAP2 binding sites (MTs) are likely to be abundant enough to capture the estimated level of expressed MAP2. A recent paper using super-resolution microscopy indicates that there could be even more MTs in dendrites than our estimate (23). This may be the reason why we did not see any changes in endogenous MAP2 despite overexpression of GFP-MAP2C, and that overexpressed MAP2C was retained in the somatodendritic region despite co-overexpression of Tau (see Fig. 1*A*).

We have previously shown that most of overexpressed Tau could bind to MTs and therefore exhibited very slow recovery in FRAP, when the phosphorylation sites in the proline-rich region (PRR) of Tau were mutated to alanine to mimic dephosphorylation (5). We also found that MT-binding of wild-type Tau is quite dynamic like that of MAP2C with faster recovery than the alanine mutant, and that pseudo-phosphorylation mutant with glutamate instead of alanine substitutions exhibited recovery even faster than wild-type Tau (5). Furthermore, our recent study suggests that the total number of non-phosphorylated sites in the PRR is more critical for stronger MT-binding of Tau than the status of specific sites in the PRR (43). These results indicated that wild-type Tau is phosphorylated at certain residues in the proline-rich region and thus exhibits dynamic MT-binding in neurons. Based on these findings, we speculate that MAP2 is also phosphorylated at certain residues in neurons (44, 45), and that the total lack of phosphorylation in *in vitro* assays results in the difference observed (44).

Is the dynamic MT-binding beneficial? For MAP2 localization, it may be. Instead of being permanently sequestered on microtubules, the dynamic binding allows MAP2 to be intermittently captured by dynein for dendritic transport. This balance between stability and mobility is likely essential for the active maintenance of its somatodendritic distribution, as demonstrated by the mislocalization of the ΔSP mutant. Another consideration is that longer residence of MAP2 on MTs may interfere with motor processivity. MAP2 has been shown to block the path of kinesin motors in *in vitro* assays (46, 47, 48, 49, 50). If this is also the case in neurons, lingering MAP2 on MTs may similarly inhibit motor-based transport and possibly its own transport. We are currently not able to implement fiber representation of MTs in the simulation. Further studies are needed to address this issue.

### Other models for the somatodendritic localization of MAP2

As explanations for the somatodendritic localization of MAP2, there have been essentially four models as described above. We cannot fully exclude the possibility that PD affects localization of MAP2. However, we showed that MAP2C natively lacking PD still exhibited somatodendritic localization, indicating that PD is not necessary for the somatodendritic localization of MAP2.

Diffusion barrier for cytoplasmic proteins like MAP2 is much less known, unlike for membrane proteins (51, 52) and the corresponding physical entity is difficult to comprehend. For MAPs, reinforced MT-binding in the AIS could make overall diffusive redistribution slower. Our FRAP in the first 50 μm of the axon and dendrites to see differences in recovery showed no differences between them (Fig. 2*B*). Although some diffusion restriction of cytoplasmic proteins has been reported (53), at least for MAP2 localization, diffusion barrier does not seem to be a critical factor. Furthermore, the AIS remained intact in ΔSP-expressing neurons despite clear axonal MAP2 mislocalization (Fig. S6*B*), arguing against the possibility that disruption of the AIS-based barrier underlies the observed mislocalization.

Regarding preferential binding of MAP2 to dendritic MTs, our FRAP experiments suggest that MT-binding is comparable in dendrites and the axon. Although some posttranslational modifications such as acetylation differ between the axon and dendrites in very immature neurons (54), they are more uniformly present in these two compartments after 7 DIV (unpublished observations), which we used in the study. Furthermore, MAP2B, which appeared to bind to MTs more stably than MAP2C does, is still mislocalized to the axon, when the motor is inhibited. These findings indicate that the preferential binding model is insufficient.

In contrast, the local synthesis and degradation model may be sufficient for MAP2 localization, when they are combined and precisely balanced. However, we (this paper) and others have shown that expressed MAP2 without any native mRNA structures such as a 3′-untranslated region, which are typically necessary for mRNA transport and local translation (55), still localizes properly to dendrites (11). Therefore, local translation of MAP2 in dendrites may not be necessary for somatodendritic localization of MAP2.

In summary, our findings strongly suggest that active transport of MAP2 by dynein is a key mechanism maintaining its somatodendritic localization. However, some critical questions remain unanswered. For example, are both axonal removal and dendritic anterograde transport of MAP2 required for somatodendritic localization of MAP2? We have not yet experimentally demonstrated the retrograde transport of MAP2 in the axon, due to insufficient levels of Dendra2-MAP2 present for photoconversion experiments. Another important question is the identity of the adaptor protein bridging the S/P region and the dynein complex, which will also help clarify whether dynein transports free MAP2 directly or as part of a MAP2-microtubule complex. Furthermore, whether the inhibition of tubulin acetylation compromises MAP2 localization remains an intriguing question. Future studies focus on clarifying these factors.

## Experimental procedures

### Neuronal culture

Dissociated cultures of embryonic (E17–18) hippocampal neurons were prepared from female timed-pregnant Sprague-Dawley rats as previously described (56) with minor modifications. Briefly, dissected hippocampi were digested in 0.25% trypsin for 15 min at 37 °C, dissociated by pipetting, and then plated onto glass coverslips coated with 1 mg/ml poly-L-lysine at 50,000 cells/coverslip. Neurons were cultured for 3 to 24 days *in vitro* (DIV) in 6-well plates containing astrocyte cultures on the bottom. Coverslips were lifted with wax pedestals as described by Kaech and Banker (57). Cytosine arabinofuranoside was added to the culture at 2 DIV to prevent the growth of non-neuronal cells on the coverslips. All animal use was approved by the institutional animal care and use committee of Doshisha University.

### DNA plasmids

Human MAP2C cDNA tagged with 6xHis was inserted into pAcGFP (Takara) vector using the In-Fusion cloning kit (Takara). MAP2C ΔSP was created by removing the “S/P region” (P134-P251 in the numbering of the 471-amino-acid isoform, MAP2C) from pAcGFP-His-MAP2C by PCR. MAP2C Δ1-308 was created by removing amino acids 1 to 308 from pAcGFP-His-MAP2C by PCR. MAP2C Δ1-308 + SP was created by amplifying the S/P region, which was inserted into pAcGFP-His-MAP2C Δ1-308 by In-Fusion cloning kit (Takara). pAcGFP-His-MAP2C-Tau was a chimera of MAP2C_M1-L311 and Tau0N4R_P193-L383 in the Tau0N4R numbering, which was created by PCR and the chimera MAP2C-Tau was amplified by PCR and inserted into the pAcGFP vector using the In-Fusion cloning kit. Dendra2-MAP2C was prepared as follows. pAcGFP-His-MAP2C without AcGFP was amplified and Dendra2 was amplified from pDendra2-C vector (Takara). Dendra2 was inserted in place of AcGFP by In-Fusion cloning kit. Dendra2-MAP2C ΔSP was prepared by removing the S/P region from Dendra2-MAP2C by PCR. MAP2B cDNA was amplified from a human hippocampus cDNA library and inserted in place of MAP2C in pAcGFP-His-MAP2C. To express shRNA targeting rat DHC1, rat DHC1-targeting oligonucleotide, AATTCAAGCTGGCGTGCAA, was inserted into *Bgl*II and *Hin*dIII site of pSUPER-retro-neo-GFP vector according to the manufacturer's manual. All constructs were sequenced to confirm the sequences. DHC1 plasmid was kindly gifted by Dr. Yoko Toyoshima (The University of Tokyo), and we inserted a Flag tag into streptavidin binding peptide tag, which is located in the N-terminal region of the plasmid. pFUGW-mCherry, pFUGW-mCherry-MAP2C WT and pFUGW-mCherry-MAP2C ΔSP was created by In-Fusion from pFUGW-GFP, a gift from Dr. Shigeo Takamori (Doshisha University).

### Transfection

Neurons were transiently transfected at 7 DIV using Lipofectamine 2000 transfection reagent (Thermo Fisher Scientific, Denmark). Neurons were transfected in separate dishes with 0.5–1 μg cDNA per coverslip. HEK293 cells (our laboratory stock) were transfected with indicated plasmids using Lipofectamine 3000 or 2000 according to the manufacturer's instructions.

### Lentiviral production

HEK293T-LentiX cells (our laboratory stock) were seeded at 1.0 × 10^6^ cells in a 10-cm dish 1 day before transfection and co-transfected with 17 μg of pFUGW-mCherry, -mCherry-MAP2C WT, or -mCherry-MAP2C ΔSP and packaging plasmids (10 μg pCAG-kGP1, 5 μg pCAG4-RTR2 and 5 μg pCAG-VSVG) by calcium phosphate transfection. After changing the medium the next day, lentiviral supernatant was harvested 2 days after transfection. The supernatant was filtered with a 0.45 μm filter and stored at −80 °C.

### AAV vector production

AAVpro 293T cells (Takara, Cat. No. 632237) were seeded at 4.2 × 10^6^ cells in a 10-cm dish 1 day before transfection. For the donor virus, 3.1 μg of plasmid containing sgRNA against rat *Tubb3* and DRSR2-linker-EGFP-DRSR2, 6.9 μg of pHelper and 5.1 μg of pUCmini-iCAP-PHP.S capsid plasmid were mixed in 900 μl Opti-MEM together with PEI (1 mg/ml). For the SpCas9 virus, 4.1 μg of px551, 6.9 μg of pHelper and 5.1 μg of pUCmini-iCAP-PHP.S capsid plasmid were mixed in the same way. Each DNA-PEI mixture was vortexed for 5 s, incubated at room temperature for 10 min and added to AAVpro 293T cells in a 10-cm dish. The next day, the medium was replaced with fresh DMEM. Three days after transfection, cells were harvested in a 1.5 ml tube by treating with prewarmed PBS containing 0.5 M EDTA for 5 min at room temperature. After centrifugation at 2000 × *g* for 5 min, the supernatant was discarded, and the pellet was centrifuged again at 2000 × *g* for 1 min to remove residual supernatant. The pellet was resuspended by vortex for 15 s, followed by addition of 1 ml of citrate buffer and incubation at 37 °C for 5 min. After brief vortexing and centrifugation at 10,000 × g for 10 min at 4 °C, the supernatant was collected in a new tube. To the supernatant, 111 μl of 2 M Tris-HCl (pH 9.5) was added. The virus-containing solution was then loaded onto an Amicon Ultra centrifugal filter (100 kDa) and centrifuged at 10,000 × *g* for 20 min.

### Generation of EGFP-tubulin knock-in neurons expressing mCherry-MAP2C

To generate EGFP-βIII-tubulin knock-in neurons, we used a CRISPR/Cas9-based, homology-independent targeted integration strategy at the C-terminus of the rat *Tubb3* locus. The genomic locus was cut using the sgRNA 5′-GATGTATGAAGATGACGACG-3′. SpCas9 was expressed from an AAV-PHP.S vector under the neuron-specific Mecp2 promoter, and the rat *Tubb3* sgRNA was driven by a hU6 promoter. The donor AAV-PHP.S vector carried an EGFP coding sequence preceded by a (GGGGS)_3_ linker. To enable Cas9-mediated cleavage of the donor, the EGFP cassette was flanked by donor recognition sequences (DRS), giving the structure DRS-linker-EGFP-DRS. In addition, the donor vector encoded a DRS-directed sgRNA under the control of an mU6 promoter. Thus, in the presence of SpCas9, the rat *Tubb3* sgRNA induces a double-strand break at the endogenous *Tubb3* locus, while Cas9, directed by the DRS sgRNA, cleaves the donor vector at the flanking DRS sites, allowing integration of the linker-EGFP cassette into the *Tubb3* locus *via* non-homologous end joining. Rat primary neurons were transduced with AAV vectors at 0 DIV. At 4 DIV, the EGFP-knock-in neurons were further transduced with lentivirus to express mCherry, mCherry-MAP2C WT or mCherry-MAP2C ΔSP for FRAP experiments.

### Immunofluorescence staining and analysis

Neurons were fixed in 4% paraformaldehyde/PBS for 20 min. Blocking and permeabilization were done in 4% dried milk/0.1% Triton X-100/TBS. Primary and secondary antibodies were diluted in the same buffer and applied in separate incubation steps of 1 h and 45 min, respectively. Coverslips were mounted on glass microscope slides using ProLong Diamond anti-fade reagent (Thermo Fisher Scientific). We used the following primary antibodies (and dilutions): anti-GFP (mouse monoclonal, 1:1000, MBL, D153), anti-DHC1 (1:1000, Proteintech, 12345-1-AP), anti-AnkyrinG (1:1000, mouse monoclonal, NeuroMab, N106/36), anti-acetylated tubulin (1:5000, Sigma, 6-11B-1), anti-MAP2 (1:1000, Sigma, AP20), anti-pan-MAP2 (1:1000, MAP2N) (58), anti-pan-sodium channel (1:500, NeuroMab, K58/35.4) and anti-MAP2 (1:2000, Biosensis, C1382-50). The specificity of the anti-DHC1 antibody was confirmed by the marked reduction of immunostaining signal in DHC1 knockdown neurons (Fig. 6*C*). Other antibodies are well-characterized and showed staining patterns consistent with their previously reported localizations.

### Live-cell imaging

For fluorescence recovery after photobleaching (FRAP) and photoconversion experiments, neurons expressing either GFP-tagged or Dendra2-tagged proteins were imaged using an Olympus FV-1000 microscope, Carl Zeiss LSM 700, or Leica SP8. Images were taken every second. Photobleaching was induced by applying the 405 nm laser to a circular (3 μm diameter) spot at 100% laser power. Laser power was reduced to 2–5% for photoconversion to avoid bleaching. Fluorescence intensity was measured using ImageJ, corrected for background fluorescence and the overall bleaching due to imaging, and normalized for the maximum and minimum fluorescence intensities.

### Dynein inhibitor

Cytoplasmic dynein inhibitor, ciliobrevin D, was purchased from Calbiochem. Neurons were treated with 50 μM ciliobrevin D for 48 h. Concentration of ciliobrevin D, 50 μM, was based on previous literature that reported higher concentration, 100 μM, also inhibited kinesin-1 and kinesin-5 motor activity (18). The effect of ciliobrevin D on the localization of MAP2 was quantified by taking the ratio of the signal intensity in the distal axon (region distal to the AIS) to that in the proximal axon (region proximal to the AIS).

### Analysis of localization of MAP2C

MAP2C localization was analyzed as follows.

Signal intensities of one of the dendrites and the axon were measured in both green (endogenous) and red (exogenous) channels using ImageJ. The selected area of the axon was 168 ± 10 μm away from the cell body. The axon/dendrite ratio was calculated as follows to normalize to the endogenous MAP2 intensity in the axon.

$$\frac{Axon}{Dendrite}=\frac{exogenous\;axon/exogenous\;dendrite}{endogenous\;axon/endogenous\;dendrite}$$

### Immunoprecipitation

Cell lysates were prepared from transfected HEK293 cells 2 days after transfection. Cells were lysed with lysis buffer, 50 mM Hepes (pH 7.4), 100 mM NaCl, 2 mM EDTA, 0.5% Triton X-100, 1 mM NaF and protease inhibitor cocktail (Roche, 04693132001). After centrifugation at 4 °C, 15,000 × *g* for 10 min, supernatants were collected as cell lysates. Protein concentration was determined by protein assay BCA kit (Nacalai, 06385-00). Immunoprecipitation was performed using Dynabeads Protein G (Invitrogen) according to the provided manual. Briefly, anti-His antibody (D291-3, MBL) was incubated with Dynabeads Protein G to bind the antibody and 500 μg lysate was added to the antibody-bound beads. After 1 h incubation at 4 °C, beads were washed with lysis buffer without NaF and protease inhibitor cocktail 3 times. Beads were transferred to new tubes and eluted with 50 μl of 1× sample buffer. Immunoprecipitated DHC1 was quantified using ImageJ.

Mouse brain lysate was prepared and subjected to co-immunoprecipitation as described below. Three-month-old female mouse was sacrificed by cervical dislocation and brain tissue was dissected. Dissected brain was homogenized with a glass-teflon homogenizer in ice-cold microtubule stabilizing buffer, MSB; 0.1 M MES (pH6.8), 10% glycerol, 1 mM MgSO_4_, 1 mM EGTA, 0.1 mM DTT, 0.5% Triton X-100, 10 mM taxol, 2 mM GTP, 0.1 mM PMSF, 0.1 mM DIFP, 1 mg/ml pepstatin, 1 mg/ml antipain, 10 mg/ml aprotinin, 10 mg/ml leupeptin, 50 μg/ml TLCK, protease inhibitor cocktail, 1 mM NaF, 1 mM β-glycerophosphate, 1 mM Na_3_VO_4_, 0.5 μM okadaic acid. Brain lysate was prepared by removing cell debris by centrifugation at 2400 × *g* for 3 min at 4 °C and subjected to coimmunoprecipitation. To perform co-immunoprecipitation, anti-MAP2 (MAP2N) and control rabbit IgG were incubated with Dynabeads Protein G for 20 min at room temperature. Antibody-bound beads were incubated with 400 μl brain lysate and 100 μl MSB(−), MSB without phosphatase inhibitors and protease inhibitors, for 30 min at 4 °C. After washing beads with MSB(−), co-IP samples were prepared by adding 1× SDS-PAGE samples buffer (2% SDS, 5% 2-mercaptoethanol, 10% glycerol, 62.5 mM Tris-HCl, pH 6.8) to the beads.

### Western blot

Proteins were separated in SDS-polyacrylamide gels and electrophoretically transferred to nitrocellulose membranes. The membranes were washed, blocked with 5% non-fat dry milk in TBST, 150 mM NaCl, 20 mM Tris-HCl (pH 7.6) and 0.05% Tween, for 30 min, and then incubated with primary antibodies overnight in TBST at 4 °C. Membranes were incubated with the following primary antibodies: anti-DHC1 (1:2000, Proteintech, 12345-1-AP), anti-GFP (1:5000, MBL, 598), anti-MAP2 (1:1000, MAP2N). The membranes were washed and probed with HRP-conjugated secondary antibodies according to the manufacturer’s instructions. Bands were detected with Immobilon Crescendo Western HRP substrate (Millipore, WBLUR0500) using LAS-4000. Immunoreactivity was quantified using ImageJ.

### Statistical analyses

All statistical analyses were performed on GraphPad Prism software (GraphPad Software, Inc., CA, USA). Data were tested for normality and lognormality using D'Agostino–Pearson, Anderson–Darling, Shapiro–Wilk, and Kolmogorov–Smirnov tests. Data that did not follow a normal distribution were analyzed using nonparametric tests (two-tailed Mann–Whitney U test for two-group comparisons; one-sample Wilcoxon signed-rank test for ratio data); lognormally distributed ratio data were analyzed with a one-sample ratio *t* test. Power analysis was performed using G∗Power (59, 60) with parameters taken from previous reports or similar experiments.

### Simulation

All simulations were performed on the Virtual Cell (VCell) platform. Models will be publicly available in the VCell database upon publication.

We started building models by estimating the concentrations of MTs (75 μM), endogenous MAP2 (5 μM), and expressed GFP-MAP2 (25 μM) based on the literature and immunofluorescence data as described in Results. Then, a cylindrical compartment (2 μm diameter, 200 μm long), in which GFP-MAP2 freely diffuses, was constructed. GFP-MAP2 in a 3 μm spot was rapidly “bleached” by converting it to non-fluorescent MAP2 in the middle of the cylinder to mimic FRAP experiments. No flux was allowed at the boundary. The time evolution of GFP-MAP2 concentration (*c*) was described by the diffusion equation with a constant diffusion coefficient (*D*).

(1) $$\frac{\partial c}{\partial t}=D\nabla^{2}c$$

Simulation was performed using the fully implicit finite volume solver with varying time steps (max 100 ms). The concentration of GFP-MAP2 in the spot was measured and plotted. To determine the diffusion coefficient of GFP-MAP2, we took advantage of our extensive FRAP data on Tau lacking the MT-binding domain, which we found to be virtually freely diffusible, and free GFP. The virtual recovery curves were fitted to the experimental data. We changed the diffusion coefficient and monitored how the sum of squares changed. This allowed us to obtain the diffusion coefficient providing the local minima in the sum of squares. The diffusion coefficient of the GFP-MAP2 was adjusted to match the data of GFP-Tau and set to 10 μm^2^/s.

Next, we implemented MT-binding reactions of endogenous MAP2 and GFP-MAP2. These reactions are reversible and generate bound species for each. MT and bound species were virtually non-diffusible, with a diffusion coefficient of 0.001 μm^2^/s. The initial condition, such as the concentration of MT-bound GFP-MAP2, was computed using a non-spatial simulation in a homogeneous environment. Then, to determine the binding kinetics, the virtual FRAP experiment was performed as described above. Here, the time evolution of GFP-MAP2 concentration was described by the reaction-diffusion equation.

(2) $$\frac{\partial c}{\partial t}=D\nabla^{2}c+R$$

For the MT binding reaction of MAP2.

(3) $$R=k_{on}\cdot \left[MT\right]\left[freeMAP2\right]-k_{off}\cdot \left[boundMAP2\right]$$

For this reaction, we set the initial *k*_on_ at 1 μM^−1^s^−1^, and *k*_off_ was adjusted to fit to the FRAP data of GFP-MAP2 to minimize the sum of squares. Then, *k*_on_ was changed from 10 to 0.01 μM^−1^s^−1^, and the best-fit *k*_on_ and *k*_off_ were searched. *k*_on_ larger than 1 μM^−1^s^−1^ provided *k*_off_ with similar fit to the values obtained at 1 μM^−1^s^−1^. The minimum *k*_on_, which provided a fit as good as with 1 μM^−1^s^−1^ or higher, was around 0.1 μM^−1^s^−1^.

To simulate the cellular transport and localization of MAP2, we replaced the cylindrical geometry with a simple model neuron. Also, instead of photobleaching, photoconversion was implemented as a conversion of green MAP2 to red MAP2 species. Furthermore, a motor species was introduced to the model to realize active transport. MAP2 species could react with MT, motor, or MT-bound motor. Only MAP2-motor-MT was allowed to undergo directional transport at 1 μm/s. The time evolution of concentrations was described by the reaction-diffusion equation, except for the MAP2-motor-MT species, of which changes were described by the reaction-diffusion-advection equation with a constant velocity of transport (*V*).

(4) $$\frac{\partial c}{\partial t}=D\nabla^{2}c-V\nabla \cdot c+R$$

The initial condition was computed using a non-spatial simulation in a homogeneous environment. The initial concentrations of MT, motor, and motor-MT were set as constants to avoid the migration of these species over time by transport (advection). MAP2 species were situated only in the soma and dendrites at the beginning.

For the stochastic simulation, we used Smoldyn, a particle-based simulator, in VCell with the parameters described above. A small cylindrical portion (5 μm × 0.2 μm × 0.2 μm) of a dendrite was simulated with a 0.01 s time step for 5 s using initial value boundary conditions. Dwell times of MAP2 on MT and motor-MT were analyzed using virtual kymographs for each species.

## Data availability

Data are available from the corresponding author upon request.

## Supporting information

This article contains supporting information.

## Conflict of interest

The authors declare that they have no conflicts of interest with the contents of this article.
