# Supplementary material for: Active transport by cytoplasmic dynein sustains dendritic MAP2 localization in developing neurons
Source: J Biol Chem. 2026 Aug 7;302(10):113407. doi: 10.1016/j.jbc.2026.113407 (PMC13571933; doi:10.1016/j.jbc.2026.113407)
Supplement: Supplementary file 1 [file mmc1.docx]

**Active transport by cytoplasmic dynein sustains dendritic MAP2 localization in developing neurons**

Yoji Yonemura^1), a),^*, Yuri Sakai^1),^*, Rinaho Nakata^1)^, Ayaka Hagita-Tatsumoto^2),b)^, Tomohiro Miyasaka^2), 3),c)^, Hiroaki Misonou^1),3)^

1) Laboratory of Ion Channel Pathophysiology, Graduate School of Brain Science, Doshisha University, Kyotanabe-shi, Kyoto, Japan

2) Department of Neuropathology, Faculty of Life and Medical Sciences, Doshisha University, Kyotanabe-shi, Kyoto, Japan.

3) Center for Research in Neurodegenerative Diseases, Doshisha University, Kyotanabe-shi, Kyoto, Japan.

a) Present address: Department of Cell Biology and Morphology, Akita University Graduate School of Medicine, Akita 010-8543, Japan

b) Present address: Department of Neuroscience, Mayo Clinic Florida, Jacksonville, Florida, USA

c) Present address: Laboratory of Physiology and Anatomy, Nihon University, School of Pharmacy, Funabashi, Chiba, 274-8555, Japan

* These authors contributed equally to this work

To whom correspondence should be addressed:

Yoji Yonemura, Department of Cell Biology and Morphology, Akita University Graduate School of Medicine, Akita 010-8543, Japan E-mail: yoji.yonemura@med.akita-u.ac.jp

Hiroaki Misonou, Laboratory of Ion Channel Pathophysiology, Graduate School of Brain Science, Doshisha University, Kyotanabe-shi, Kyoto 610-0394, Japan. E-mail: [hmisono@mail.doshisha.ac.jp](mailto:hmisono@mail.doshisha.ac.jp)

List of the materials:

Fig. S1

Fig. S2

Fig. S3

Fig. S4

Fig. S5

Fig. S6
Fig. S7

**Fig. S1. Localization of MAP2**

A, Localization of GFP-MAP2A in a 10 DIV neuron. Dashed area is magnified to show localization of MAP2A in the axon. The arrow indicates the axon. Scale bar, 20 µm.

B, Quantitative analysis of localization of rMAP2A(ratMAP2A), rMAP2B, and rMAP2C. Data are presented as mean ± SD. No statistical significance was observed among the three groups (one-way ANOVA, F(2, 30) = 2.269, *p* = 0.1209). n = 14 (MAP2A), 10 (MAP2B), 9 (MAP2C) from three independent experiments.

C, Domain structure of MAP2C used in this study. NTR: N-terminal region, NAAR: Neutral amino acids region, S/P: serine/proline rich region, TLR: Tau like region, MTBD: microtubule binding domain, CFR: C-terminal franking region, CTR: C-terminal region.

D, Overall localizations of wild-type MAP2C and 3 mutants. Lower panels are cropped images of upper panels. The arrows indicate the axons. Scale bar, 50 µm (for upper panels) and 20 µm (for lower panels).

**Fig. S2. MAP2C binding dynamics are microtubule-dependent.**

 FRAP of GFP-tagged MAP2C before and after nocodazole treatment. Neurons were imaged for FRAP (untreated) and treated with 1 µg/ml nocodazole for 10–20 min, and imaged again for FRAP (nocodazole). Data are presented as mean ± SD with n = 7 (untreated) and 6 (nocodazole) from 2 independent experiments.

**Fig. S3. Comparison of motor- and MT polymerization-based models**

Left panel shows the temporal changes of line profiles extracted from the model described in Fig. 4. Right panel is based on the model in which MAP2 moves along with polymerizing MTs.

**Fig. S4. Effects of Dynein inhibition**

A, Overall distributions of endogenous tubulin (red) and MAP2 (green) after 24 h treatment with ciliobrevin D. Scale bar, 20 µm.

B, Axonal distribution of endogenous MAP2 (green) in a neuron treated with ciliobrevin D. AIS (red) is indicated by staining with pan-sodium channel antibody. Scale bar, 20 µm.

C, Representative images of DMSO or ciliobrevin D treated neuron stained with anti-MAP2 (green) and anti-AnkG (red). Arrow indicates axon. Dashed area is shown in Fig. 6A. Scale bar, 20 µm.

**Fig. S5. Effects of Dynein knockdown**

A, Knockdown of endogenous DHC1 with shRNA. Arrow indicates transfected neuron. Neurons were transfected with shRNA at DIV6 or 7 and fixed at DIV13 or 14. Scale bar, 50 µm.

B, Quantitative analysis of DHC1 immunolabeling in neurons treated with scrambled or DHC1 shRNA. n = 22 (scramble) and 15 (DHC1) from 3 independent experiments. Data are presented as mean ± SD. **p* = 0.0002 using two-tailed Mann –Whitney U test.

C, Representative images of neurons transfected with sh-scramble or sh-DHC1, stained with MAP2 and Ankyrin G. Dashed area is shown in Fig. 6D. Arrow indicates axon. Scale bar, 20 µm.

**Fig. S6. The AIS remains intact in ΔSP-expressing neurons**

A, Quantification of AnkG signal intensity at the AIS. Neurons transfected with GFP-MAP2C WT or ΔSP were immunostained with an anti-AnkG antibody. The fluorescence intensity of AIS was measured. All values are normalized to the mean AnkG intensity of the MAP2C WT-expressing neurons. Data are presented as mean ± SD, n = 8 for WT and n = 7 for ΔSP from 2 independent experiments.

B, Representative immunofluorescence images showing intact AIS structures in MAP2C ΔSP expressing neurons. Arrows indicate axon. The mislocalization of MAP2C ΔSP into the axon is shown in lower left panel (vs upper left, WT). Arrowheads point to the AIS. Note that neighboring untransfected neurons in the same imaging field show comparable AnkG signal intensities.

C, Representative immunofluorescence images showing MAP2 (endogenous) mislocalization and AIS intensity in DMSO or ciliobrevin D-treated neurons. Arrows indicate axon. Arrowheads point to the AIS.

D, Quantification of AnkG signal intensity following pharmacological inhibition of dynein. Neurons were treated with DMSO or ciliobrevin D for 48 h, fixed and stained with an anti-AnkG antibody. All values are normalized to the mean intensity of the DMSO-treated group. Data are presented as mean ± SD, n = 19 from 2 independent experiments.

E, Representative immunofluorescence images showing endogenous MAP2 localization and AIS intensity in shRNA-transfected neurons. GFP positive cells are Neuron transfected with scramble or shRNA against DHC1. While AnkG signal intensity in scramble-transfected neurons was comparable to that in neighboring untransfected neurons, shDHC1-expressing neurons exhibited a noticeable reduction in AnkG signal compared to adjacent untransfected controls. Arrows indicate axon. Arrowheads point to the AIS.

F, Quantification of AnkG signal intensity following DHC1 knockdown. Neurons were transfected with scramble or DHC1-targeting shRNA, fixed and stained for AnkG. All values are normalized to the mean intensity of the control group (scramble). Data are presented as mean ± SD (n = 12 for scramble and 6 for shRNA targeting DHC1) from 2 independent experiments.

**Fig. S7. Co-Immunoprecipitation shows that MAP2 interacts with DHC1 through the S/P region**

A and B, Mouse brain lysates were incubated with indicated antibodies with magnetic beads and immunoprecipitates were subjected to immunoblot analysis with anti MAP2 antibody (A) or anti DHC1 antibody (B). Rabbit IgG was used as negative control.

C and D, HEK 293 cells were transfected with DHC1 and His-tagged GFP 2x, GFP-MAP2CWT or GFP-MAP2CΔSP. Lysates were immunoprecipitated with anti His antibody. Cell lysate (input) and Immunoprecipitates (IP) were subjected to immunoblot analysis with antibody against GFP (C) and DHC1 (D). GFP 2x was used as negative control.
